# Left ventricular diastolic dysfunction index based on non-invasive measurements

**DOI:** 10.1186/1532-429X-17-S1-Q24

**Published:** 2015-02-03

**Authors:** Chun G Schiros, Thomas S Denney, Inmaculada Aban, Himanshu Gupta

**Affiliations:** 1 University of Alabama at Birmingham, Birmingham, AL, USA; 2 Auburn University, Auburn, AL, USA

## Background

Echocardiographic tissue Doppler or magnetic resonance imaging (MRI) measurements of early diastolic mitral annular velocity with other appropriate parameters are frequently used as a non-invasive diagnostic tool of diastolic dysfunction. Previously published global approach utilizing normalized left ventricular (LV) torsion shear angle volume loop ( *φ_hat V_hat* loop) was proposed to provide a new global description of LV diastolic function. The purpose of this study was to evaluate the discriminant power of these non-invasive parameters in identifying elevated LV end-diastolic pressure (LVEDP) (i.e. LVEDP≥15ml) and provide a non-invasive index to predict elevated LVEDP.

## Methods

A group of 23 patients with LV ejection fraction≥50% without acute infarct undergoing cardiac catheterization that did not undergo percutaneous coronary intervention were studied using high-fidelity pressure measurement. Echocardiogram with flow and tissue Doppler quantification was performed on the same date of cardiac catheterization for all participants. Cine and tagged cardiac MRI were performed on all subjects, followed by comprehensive volumetric and strain analysis. The database consisted of five parameters collected from all subjects: torsion hysteresis area (THA), peak -d*φ_hat*/d*V_hat* at early diastole, MRI derived E/A_MRI_, echocardiographic derived E/A and E/e'. Stepwise variable selection was applied to select parameters with significance level of leave out and stay in equal to 0.15. A logistic regression classifier was used to construct the non-invasive index for identifying elevated LVEDP based on the selected parameters. The classifier's prediction performance was analyzed using a Receiver-Operating Characteristic (ROC) curve and expressed as its sensitivity, specificity, accuracy and area under the curve (AUC).

## Results

**Figure 1:**
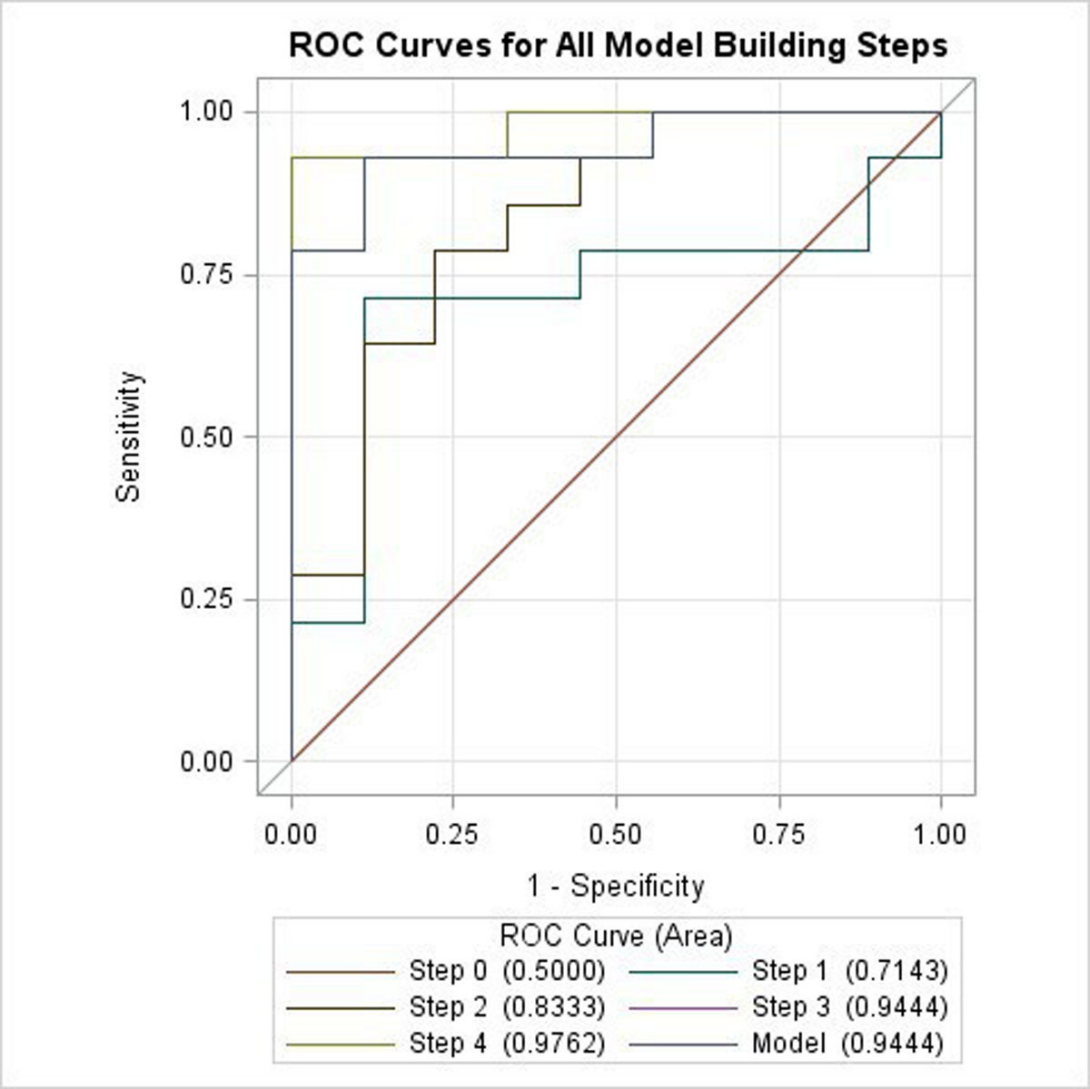
ROC curves for all model building steps. Step 0 contains only the intercept; step 1 selects peak -dφ_hat/dV_hat at early diastole in the model; step 2 adds torsion hysteresis area into the model; step 3 adds E/A ratio into the model while step 4 adds mean E/e' into the model. The Wald chi-square statistics determines to remove mean E/e'. The final model therefore contains parameters peak -dφ_hat/dV_hat at early diastole, torsion hysteresis area and E/A.

Among all parameters, peak -d*φ_hat*/d*V_hat* at early diastole, has the highest chi-square score of 2.45 (p=0.12), indicating it as the best discriminator compared with others. All other variables had chi-square score<1 and p value>0.4. Stepwise selection chose peak peak -d*φ_hat*/d*V_hat* at early diastole, at early diastole, THA and echo measured E/A to construct the logistic regression model (Table 1, Figure 1). The model predicted elevated LVEDP with sensitivity of 93%, specificity of 89%, accuracy of 91%, and AUC of 0.94 (Figure 1).

**Table 1:** Summary of stepwise selection

Step	Variable		Degrees of Freedom	Number In	Chi-Square Score	Wald Chi-Square	P value
						
	Entered	Removed					
1	Peak -dφ_hat/dV_hat		1	1	2.45		0.1174

2	Torsion Hysteresis Area		1	2	5.32		0.0211

3	E/A		1	3	5.08		0.0242

4	Mean E/e'		1	4	2.66		0.1031

5		Mean E/e'	1	3		1.29	0.2552

## Conclusions

MRI derived global parameter peak -d*φ_hat*/d*V_hat* at early diastole was the best discriminator compared with other non-invasive measures in identifying elevated LVEDP. Moreover, by combining THA, peak -d*φ_hat*/d*V_hat* at early diastole and echo measured E/A through stepwise selection, the logistic regression model can identify LVEDP≥15ml with great accuracy, indicating that these parameters are determined by different factors and together, they are able to predict diastolic dysfunction non-invasively.

## Funding

NIH NHLBI R01-HL104018.

